# A Self-Powered Engine Health Monitoring System Based on L-Shaped Wideband Piezoelectric Energy Harvester

**DOI:** 10.3390/mi9120629

**Published:** 2018-11-28

**Authors:** Shuxin Shi, Qiuqin Yue, Zuwei Zhang, Jun Yuan, Jielin Zhou, Xiaokun Zhang, Shan Lu, Xin Luo, Chongyu Shi, Hua Yu

**Affiliations:** 1 Key Laboratory for Optoelectronic Technology & Systems, Ministry of Education of China, Key Laboratory of Fundamental Science of Micro/Nano-Device and System Technology, Chongqing University, Chongqing 400044, China; 20160802007t@cqu.edu.cn (S.S.); vinicolor.violet.end@gmail.com (J.Z.); 20160813100@cqu.edu.cn (X.Z.); 20103160@cqu.edu.cn (S.L.); 201708131108@cqu.edu.cn (X.L.); 201708021004@cqu.edu.cn (C.S.); 2 Department of Electro-Mechanic Engineering, Chongqing College of Electronic Engineering, Chongqing 401331, China; yqq622@163.com; 3 Chongqing Acoustic-Optic-Electric Corporation, China Electronic Technology Group Corporation, Chongqing 400060, China; zhangzuwei00@163.com; 4 Science and Technology on Analog Integrated Circuit Laboratory, Chongqing 401332, China; yjwlw@semi.ac.cn

**Keywords:** engine health monitoring, L-shaped wideband vibration energy harvester, power management, wireless sensors nodes

## Abstract

Engine health monitoring is very important to maintain the life of engines, and the power supply to sensor nodes is a key issue that needs to be solved. The piezoelectric vibration energy harvester has attracted much attention due to its obvious advantages in configuration, electromechanical conversion efficiency, and output power. However, the narrow bandwidth has restricted its practical application. A self-powered engine health monitoring system was proposed in this paper, and an L-shaped wideband piezoelectric energy harvester was designed and implemented. The L-shaped beam-mass structure and the piezoelectric bimorph cantilever beam structure was combined to form the wideband piezoelectric energy harvester configuration, which realized effective output at both resonance points. The theoretical model and finite element simulation analysis of the wideband piezoelectric energy harvester were proposed and the parameters were optimized based on that to meet the requirement of the vibration frequency of the engine. The experimental results show that the proposed energy harvester can be applied into the automobile engine health monitoring system. Engine signal analysis results also demonstrate that the proposed system can be used for engine health monitoring.

## 1. Introduction

As the most common power mechanical equipment, engines are widely used for automobiles, ships and aviation aircraft. The engine’s reliability directly affects the operation security of the system. To enhance the operation reliability of repair and maintenance, continuous real-time monitoring of the operating state of the engine is particularly important. A convenient and reliable solution is to use the wireless acceleration sensor nodes to sense the vibration frequency of engines. However, due to the batteries needing to be replaced or charged periodically, which increases difficulty of maintenance, finding a non-battery power supply way for the wireless acceleration nodes is a key issue to push the application of the engine health monitoring technique. Due to there being a lot of energy in the environment, exploring the technology of harvesting energy from the environment has important significance [1,2,3]. A self-powered monitoring and sensing system based on a vibration energy harvester has emerged as one of the most attractive alternative systems [4,5,6,7].

There has been a lot of work dedicated to developing wireless sensing-based structural monitoring systems. In [8], a piezoelectric energy harvester based on a novel analog control unit was proposed. Through simulations and experiment, the maximum transferred power from the piezoelectric generator to load was ensured along wide range load conditions. The results of the research were applied to the structural health monitoring of critical components of the aircraft. A self-powered wireless bridge health monitoring system was proposed in [9]. This study proposed the entire system from power supply to data transmission, and the experimental results showed that the piezoelectric energy harvester could support the system. Although the piezoelectric energy harvester has the obvious advantage in electromechanical conversion efficiency and output power, as a result of relying on resonance, the narrow bandwidth makes the piezoelectric vibration energy harvester unable to thrive in a wideband engine vibration environment [10]. Certainly, some efforts have been made to broaden the bandwidth of the piezoelectric vibration energy harvesters. In [11], an amplitude limiter-based piezoelectric energy harvester was designed. The prototype realized not only a 22 Hz operation bandwidth, but also additional significant output power. Tanju Yildirim et al. [12] designed a motion-based energy harvester (MBEH) based on the nonlinear response of a parametrically excited cantilever beam in combination with mechanical stoppers. Results showed that the energy harvesting bandwidth was increased in the range of 35–145%. Thomas Searl et al. [13] designed a coupled parametric-transverse nonlinearly broadband energy harvester utilizing mechanical stoppers, as a result, the nonlinear energy harvested by the system takes place over a much broader-frequency bandwidth. There are also many studies on the energy management circuit of piezoelectric energy harvesters. In [14], a novel piezoelectric harvester-based self-powered adaptive circuit with wireless data transmission capability for structural health monitoring is presented. The circuit can work with different piezoelectric ceramic transducers (PZT) and different load conditions and is able to accomplish the maximum power transferred condition along different amplitudes and frequencies of oscillations of the PZT. However, few of these works were designed based on actual application scenarios.

This paper aims to provide a self-powered solving plan for the engine health monitoring and explore the bandwidth expansion technology of the piezoelectric energy harvesters aiming at the wideband engine vibration. Firstly, the paper measured and analyzed the basic characteristics of engine vibration signal taking the Chevrolet Epica 2HO automotive engine. Secondly, the self-powered system for engine health monitoring was proposed based on the application environment of the automobile engine. Then the paper developed the wideband piezoelectric energy harvester. The finite element analysis of the designed wideband piezoelectric vibration energy harvester were present to define the optimal parameters. Finally, the test experiment system was set up and implemented and the analysis results of the signals were provided.

## 2. Theoretical Analysis of Engine Vibration Signal

The engine is a complicated mechanical system and according to statistics, more than 90% of engine structural failures are caused by vibration or related to vibration of engines. There are many kinds of exciting forces that cause engine vibration. In reality, the exciting force that causes the engine vibration to increase is mainly the unbalanced force of the rotating member and the exciting force of the gas. The components where the engine fails are mainly the rotors, bearings, and blades. According to the vibration phenomenon, the vibration fault is roughly divided into: vibration only in a certain speed range; vibration in several speed ranges; and vibration during acceleration.

**Figure 1:**
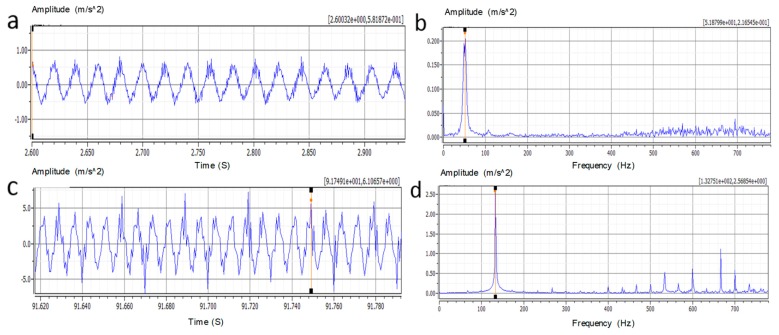
(**a**,**b**) The vibration signal in Z direction on the cylinder head of the 2HO engine at 800 r/min in time and frequency domain; (**c**,**d**) The vibration signal in Z direction on the cylinder head of the 2HO engine at 4000 r/min in time and frequency domain.

For convenience, this paper take the Chevrolet Epica 2HO automotive engine (Chevrolet, Detroit, MI, USA) as the monitoring object and recorded the vibration signals on the cylinder head in both time and frequency domains. Vibration signals were collected in normal and no-load running conditions and varied from seven different speeds, as summarized in Table 1. All the data were taken from the *z*-axis, which is normal to the deck, with the highest average acceleration level that would give the maximum output power from a piezoelectric energy harvester. Besides, the plots of vibration signal at the lowest speed of 800 r/min and highest speed of 4000 r/min are given in Figure 1.

Although the time domain waveforms of vibration signals in Figure 1a,c is bewildering, periodic gas force excitation can be observed obviously. The Fast Fourier Transform was used to analyze the frequency domain characteristic information of the vibration signal, the results demonstrate the gas force’s prompted vibration is laid on the low frequency band and the value of the frequency is apparently positively related to engine speed, which is also confirmed in Table 1. As such, these characterizations provide practical performance requirements for the design of the piezoelectric vibration energy harvester used in the particular application. The designed piezoelectric vibration energy harvester should work in the z direction, withstand accelerations up to 7 m/s^2^, and extract the electrical response in around 50 to 130 Hz frequency range.

## 3. The Proposed Self-Powered Engine Health Monitoring System

**Figure 2:**
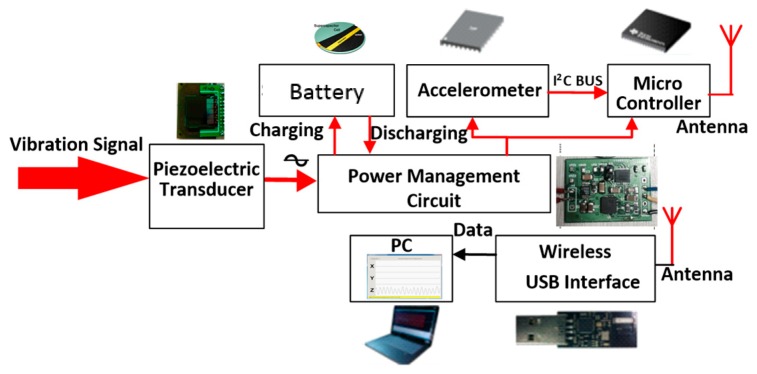
The proposed self-powered engine health monitoring system.

The scheme of the proposed self-powered engine health monitoring system in this paper is shown in Figure 2. The system consists of two main parts: a self-powered vibration sensor node and a PC terminal. The self-powered sensor node is composed of a piezoelectric energy harvester, a power management circuit, an energy storage device, a low power accelerometer, a CC430 wireless MCU (Texas Instruments, Dallas, TX, USA), and an antenna. The piezoelectric energy harvester scavenges energy from vibration environment, and converts it into electrical energy. The power management circuit is used for impedance matching of the energy harvester and to improve the energy conversion efficiency, it can store the energy in a super capacitor and supply power to the load through an instantaneous leakage circuit. The acceleration sensor senses the vibration signal of the engine and digitizes the acquired signals. The CC430 is used for simple signal analysis, packing, and fusion of coherent data. It can reduce the complexity of data transmission and realize low power optimization of sensor nodes. Then the data is stored and sent to the wireless data transmission unit via Simplici TI protocol. The PC terminal is comprised of a wireless network access node based on the CC430 wireless-USB interface and a PC data processing software. The wireless data transmission interface is used for transmitting, receiving and managing data and the software is used to process the vibration signal.

### 3.1. Design of the Wideband Piezoelectric Energy Harvester

#### 3.1.1. Structural Design and Theoretical Model

**Figure 3:**
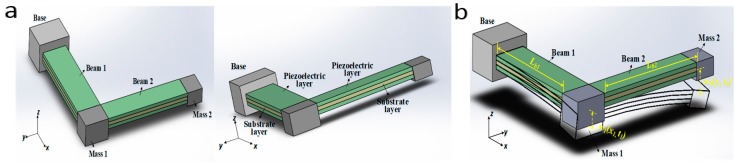
(**a**) The schematic of the wideband piezoelectric vibration energy harvester (**b**) The bending vibration mode of the wideband piezoelectric vibration energy harvester.

Considering the 80 Hz frequency range of the monitored engine vibration environment, this paper combined the L-shape beam-mass structure and the piezoelectric bimorph cantilever beam structure to form the wideband piezoelectric energy harvester configuration, as shown in Figure 3a. By the advantage of tuning to the first two natural frequencies relatively close to each other, the L-shape beam-mass structure can be used as a wideband energy harvester.

Specifically, the wideband piezoelectric vibration energy harvester is composed of the beam-mass structure in the x direction (referred to as ‘Beam_1-Mass_1’) and the beam-mass structure in the y direction (referred to as ‘Beam_2-Mass_2’). Each of the beams has one substrate layer in the middle and two piezoelectric layers in the above and below, which are connected electrically in series respectively. And all these layers are geometrically uniform along their longitudinal directions. The wideband piezoelectric vibration energy harvester is excited by the vertical base acceleration in the z direction and the two beams separately generate electrical energy through the transverse mode (d31) piezoelectric effect.

To simplify the analysis, two conditions are assumed during the mathematical modeling: (1) The effects of shear deformation and rotary inertia are neglected and each beam in the one dimensional can be looked as an Euler–Bernoulli beam with lumped mass and; (2) The two beams are supposed to join rigidly to each other and each of them can be regarded as an cantilever beam with lumped mass.

As shown in Figure 3b, considering the linearly dynamic behavior and the bending vibration modes of the wideband piezoelectric vibration energy harvester, then the undamped free vibration equation of each beam in its lateral direction can be written as:

(1) $$EI_{1}\frac{\partial^{4}w_{1}\left(x_{1},t\right)}{\partial x_{1}^{4}}+m_{1}\frac{\partial^{2}w_{1}\left(x_{1},t\right)}{\partial t^{2}}=0$$

(2) $$EI_{2}\frac{\partial^{4}w_{2}\left(y_{2},t\right)}{\partial y_{2}^{4}}+m_{2}\frac{\partial^{2}w_{2}\left(y_{2},t\right)}{\partial t^{2}}=0$$

where, $w_{1}\left(x_{1},t\right)$ and $w_{2}\left(y_{2},t\right)$ denote as the relative vertical displacements of the Beam_1 along the *x*-axis and the Beam_2 along the *y*-axis, E*I*_1_ and E*I*_2_ correspond to the equivalent stiffness of the Beam_1 and Beam_2, *m*_1_ and *m*_2_ are the mass per unit length of the Beam_1 and Beam_2, respectively.

To solve this fourth order coupled partial differential equations, a total of boundary conditions and transition conditions are required.

At $x_{1}=0$, where the Beam_1 is fixed to the base, the displacements and deflection angles of the Beam_1 are equals to 0:

(3) $$w_{1}\left(0,t\right)=0\;{\frac{\partial w_{1}\left(x_{1},t\right)}{\partial x_{1}}|}_{x_{1}=0}=0$$

At $x_{1}=L_{b1}$ and $y_{2}=0$, where the two Beams are rigid joined, the displacements and deflection angles of the two Beams are equal for all time:

(4) $$w_{1}\left(0,t\right)=0\;{\frac{\partial w_{2}\left(y_{2},t\right)}{\partial y_{2}}|}_{y_{2}=0}={\frac{\partial w_{1}\left(x_{1},t\right)}{\partial x_{1}}|}_{x_{1}=L_{b1}}=0$$

and the shear force and bending moment equilibrium relations of the Beam_1 and Beam_2 are:

(5) $${EI_{1}\frac{\partial^{3}w_{1}\left(x_{1},t\right)}{\partial x_{1}^{3}}|}_{x_{1}=L_{b1}}=\left(M_{1}+m_{2}L_{b2}+M_{2}\right){\frac{\partial^{2}w_{1}\left(x_{1},t\right)}{\partial x_{1}^{2}}|}_{x_{1}=L_{b1}}$$

(6) $${EI_{2}\frac{\partial^{2}w_{2}\left(y_{2},t\right)}{\partial y_{2}^{2}}|}_{y_{2}=0}={EI_{1}\frac{\partial^{2}w_{1}\left(x_{1},t\right)}{\partial x_{1}^{2}}|}_{x_{1}=L_{b1}}+J_{1}{\frac{\partial^{3}w_{1}\left(x_{1},t\right)}{\partial t^{2}\partial \;x_{1}}|}_{x_{1}=L_{b1}}$$

where $M_{1}$ and $M_{2}$ are the two lumped masses Mass_1 and Mass_2, and $J_{1}$ is the rotary inertias of Mass_1.

At $y_{2}=L_{b2}$, which is the free end of the Beam_2, the shear force and bending moment equilibrium relations are:

(7) $${EI_{2}\frac{\partial^{2}w_{2}\left(y_{2},t\right)}{\partial y_{2}^{2}}|}_{y_{2}=L_{b2}}=0$$

(8) $${EI_{2}\frac{\partial^{3}w_{2}\left(y_{2},t\right)}{\partial y_{2}^{3}}|}_{y_{2}=L_{b2}}=M_{2}{\frac{\partial^{2}w_{2}\left(y_{2},t\right)}{\partial t^{2}}|}_{y_{2}=L_{b2}}$$

The relative displacements $w_{1}\left(x_{1},t\right)$ and $w_{2}\left(y_{2},t\right)$ can be expanded into the spatial and temporal domains based on the separated-variable method:

(9) $$w_{1}\left(x_{1},t\right)=\sum_{r=1}^{\infty }\emptyset_{1r}\left(x_{1}\right)\eta_{r}\left(t\right)$$

(10) $$w_{2}\left(y_{2},t\right)=\sum_{r=1}^{\infty }\emptyset_{2r}\left(y_{2}\right)\eta_{r}\left(t\right)$$

where $\emptyset_{1r}\left(x_{1}\right)$ and $\emptyset_{2r}\left(y_{2}\right)$ refer to the mass normalized eigenfunctions of the *r*-th mode of vibration, and $\eta_{r}\left(t\right)$ is the corresponding time dependent amplitude. Specifically, they can be written as:

(11) $$\emptyset_{1r}\left(x_{1}\right)=A_{1r}\sin\alpha_{r}x_{1}+B_{1r}\cos\alpha_{r}x_{1}+C_{1r}\sinh\alpha_{r}x_{1}+D_{1r}\cosh\alpha_{r}x_{1}$$

(12) $$\emptyset_{2r}\left(y_{2}\right)=A_{2r}\sin\beta_{r}y_{2}+B_{2r}\cos\beta_{r}y_{2}+C_{2r}\sinh\beta_{r}y_{2}+D_{2r}\cosh\beta_{r}y_{2}$$

(13) $$\eta_{r}\left(t\right)=H_{r}e^{j\omega_{r}t}$$

and it is convenient to define $\alpha_{r}$ and $\beta_{r}$ as:

(14) $$\omega_{r}^{2}=\frac{\alpha_{r}^{4}EI_{1}}{m_{1}}=\frac{\beta_{r}^{4}EI_{2}}{m_{2}}$$

where, $\omega_{r}$ is the *r*-th vibration mode natural frequency of Beam_1 and Beam_2 undamped free vibration.

To find the specific solution, coefficients are determined so that all boundary and transition conditions are satisfied. Substituting (11) and (12) to (3)–(8) yields a linear system of equation in the form:

(15) KC=0

where K is a 8 × 8 matrix. In order to have a non-trivial solution, the rank of K must be equal to zero. Solving the corresponding equation for $\alpha_{r}$ and $\beta_{r}$, the undamped natural frequencies $\omega_{r}$ can be obtained from (3.15) with (3.13).

Since the rank of K is zero, there are infinitely many solutions for C. The eigenfunctions can be massed normalized to give a unique solution according to the orthogonality condition:

(16) $$\begin{array}{l} \int_{0}^{L_{b1}}m_{1}\emptyset_{1r}\left(x_{1}\right)\emptyset_{1s}\left(x_{1}\right)dx_{1}\\ \;\;\;\;\;\;\;+\int_{0}^{L_{b2}}m_{2}\emptyset_{2r}\left(y_{2}\right)\emptyset_{2s}\left(y_{2}\right)dy_{2}\\ \;\;\;\;\;\;\;+\left(M_{1}+m_{2}L_{b2}+M_{2}\right)\emptyset_{1r}\left(L_{b1}\right)\emptyset_{1s}\left(L_{b1}\right)+M_{2}\emptyset_{2r}\left(L_{b2}\right)\emptyset_{2s}\left(L_{b2}\right)\\ \;\;\;\;\;\;\;+J_{1}{\frac{d\emptyset_{1r}\left(x_{1}\right)}{dx_{1}}|}_{x1=L_{b1}}{\frac{d\emptyset_{1s}\left(x_{1}\right)}{dx_{1}}|}_{x1=L_{b1}}=\delta_{rs} \end{array}$$

where, $\delta_{rs}$ is Kronecker delta and defined as being equal to unity for r=s and equal to zero for r≠s.

From the above analysis we can see, the natural frequency of the wideband piezoelectric vibration energy harvester is mainly related to the equivalent stiffness and dimension parameters of piezoelectric bimorph cantilever Beam_1 and Beam_2 and the dimension parameters of the Mass_1 and Mass_2. The output voltage of the wideband piezoelectric vibration energy harvester at the natural frequency is mainly related to the damping ratio of the structure. The output voltage increases as the damping ratio decreases.

#### 3.1.2. Finite Element Simulation Analysis

**Figure 4:**
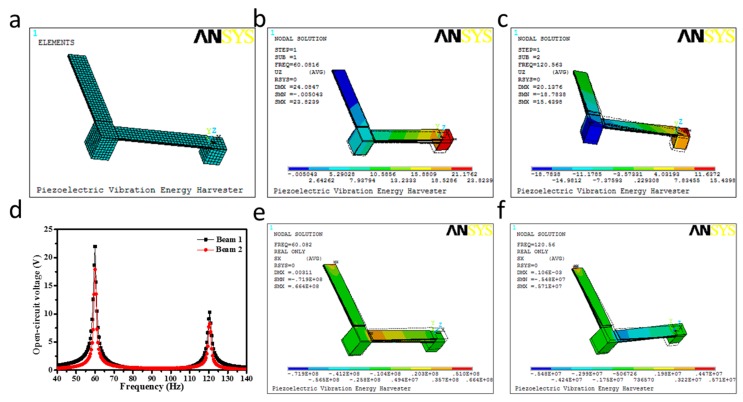
(**a**) Finite element models of the wideband piezoelectric vibration energy harvester in ANSYS; (**b**,**c**) The first two vibration modal analysis of the wideband piezoelectric vibration energy harvester; (**d**) The dynamic frequency vs. the open-circuit voltage; (**e**,**f**) The stress nephograms along the length of the beams of the wideband piezoelectric vibration energy harvester.

The prosed wideband piezoelectric vibration energy harvester was simulated using finite element method to predict the resonance frequencies and output characteristics of the harvester. The materials of the piezoelectric layer, substrate layer, and lumped mass were determined each as PZT-5H and phosphor bronze. Detailed structural parameters are listed in Table 2. Figure 4a shows the created finite element model of the harvester.

Firstly, the modal analysis was conducted to determine the resonance frequencies and corresponding vibration modes. As shown in Figure 4b,c, the first two resonance frequencies of the wideband piezoelectric vibration energy harvester are 60.082 Hz and 120.56 Hz respectively, which are laid on the 50 to 130 frequency range of the tested engine vibration environment. Secondly, the harmonic analysis was implemented to calculate the induced open-circuit voltage response of the wideband piezoelectric vibration energy harvester to harmonic excitation over a frequency range. According to the curves between the induced open-circuit voltage and the dynamic frequency in Figure 4d, both the two beam-mass structures have two peaks occurred at the first two resonance frequency points in the 50 to 130 frequency range under the 0.5 g acceleration excitation. Figure 4e,f show that the stress nephograms along the length of the two beams in the resonance are different which results in the induced open-circuit voltages of the Beam_1-Mass_1 are much larger than that of Beam_2-Mass_2.

#### 3.1.3. Dimension Optimization

**Figure 5:**
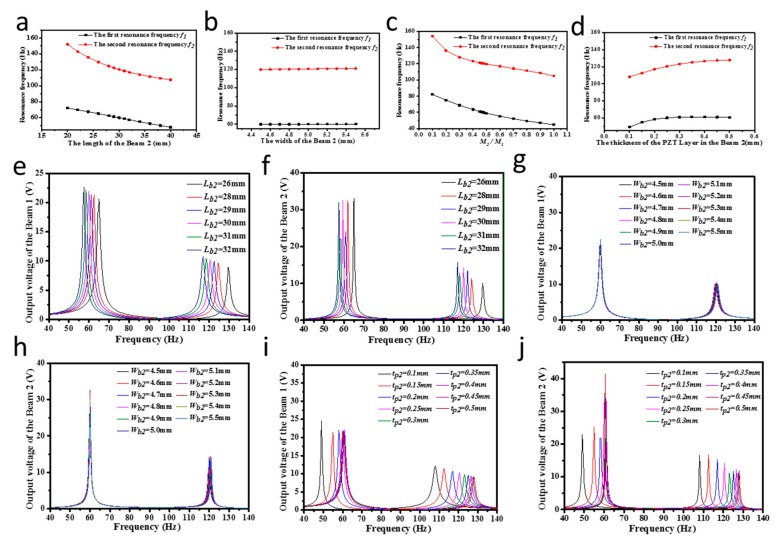
(**a**–**d**) The dimensions of the Beam_2 vs. resonance frequency; (**e**,**f**) The length of the Beam_2 vs. the output open-circuit voltage of the Beam_1 and the Beam_2; (**g**,**h**) The width of the Beam_2 vs. the output open-circuit voltage of the Beam_1 and the Beam_2; (**i**,**j**) The thickness of the PZT layer in the Beam_2 vs. the output open-circuit voltage of the Beam_1 and the Beam_2.

This paper meanly focuses on the impacts of Bema2-Mass_2 on the output performance of the proposed wideband piezoelectric vibration energy harvester using the one-variable method. Figure 5a–d presents how the resonance frequencies of the wideband piezoelectric energy harvester are affected by the length, the width, the thickness of the PZT layer and the lumped mass M2. With the increasing width of the Beam_2 or the thickness of the PZT layer in the Beam_2, there is a growth trend in the resonance frequency. However, the resonance frequency dramatically decreases as the length of the Beam_2 and the lumped mass M2 increase. Accordingly, by adjusting the length of the Beam_2, the width of the PZT layer and the lumped mass M2, the resonance frequency of the wideband piezoelectric energy harvester can be significantly changed.

Figure 5e–j show the dependence of the output open-circuit voltage on the parameters of the Beam_2. It is found that when the length of the Beam_2 is 30 mm, the width of the Beam_2 is 5 mm and the thickness of the PZT layer in the Beam_2 is 0.25 mm, both the two beams have the comparatively optimal output open-circuit voltages at resonance in the 50 to 130 Hz range. Besides, the ratio of the first two resonance frequencies of the wideband piezoelectric vibration energy harvester then equals to 2 under these specified parameters, which is well in agreement with the two-to-one internal resonance researched by Nayfeh et al. According to the above analysis results, the structural parameters of the wideband piezoelectric energy harvester in Table 2 have already been optimized.

#### 3.1.4. Fabrication Process

**Figure 6:**
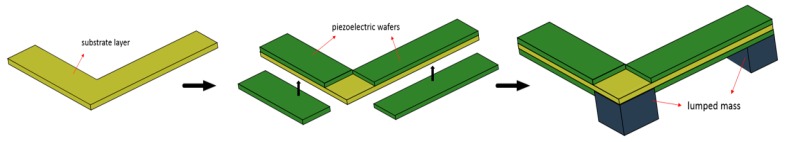
Fabrication process of the harvester.

As mentioned above, PZT-5H is used for piezoelectric ceramic plates, and phosphor bronze is used for cantilever beam and lumped mass. The fabrication process of the harvester is shown in Figure 6. First, the surface of the cantilever beam and the mass is sanded with sandpaper to provide a certain roughness for easy bonding. Then the piezoelectric ceramic plates, the cantilever beam and the mass were sequentially washed with acetone solution and ethanol solution to remove dust, oil, and oxides on their surface. Next, stick the piezoelectric ceramic plates to the surface of the cantilever beam by using epoxy based electric conduction silver gum as adhesive. They were placed in a vacuum drying oven and heated at a temperature of 60 °C for about 2 h to completely cure the electric conduction silver gum. Finally to ensure insulation between the mass and the cantilever beam, the two lumped masses were adhered to the end of cantilever beam with AB adhesive and stand for 10 min until it is completely solidified.

### 3.2. Power Management Circuit

**Figure 7:**
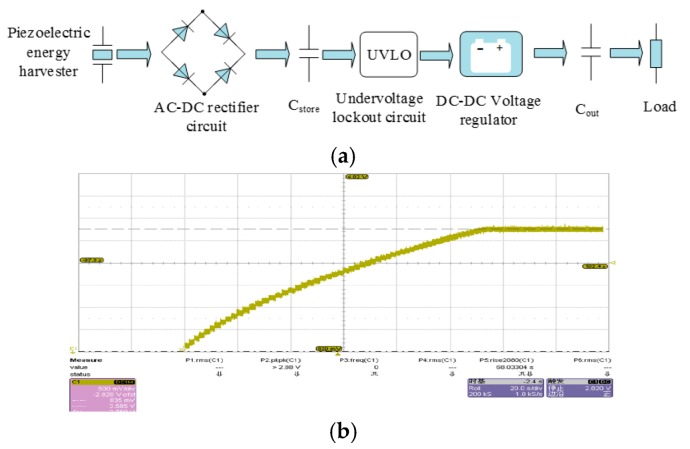
(**a**) The management circuit for piezoelectric energy harvester; (**b**) Experiment curve of charging process.

The AC voltage from the piezoelectric energy harvester could be used as a power source only through the designed power manage circuit. There are several important design factors to be considered while designing, including: (a) maximizing power extraction from energy harvester by using the impedance match technology; (b) storing and monitoring the harvested energy; (c) conditioning output voltage to meet the power supply requirements of the accelerometer; (d) reducing power consumption as much as possible. Based on the above design factors, this paper proposed a scheme of the energy management circuit as shown in Figure 7a.

The piezoelectric energy harvester converts the collectable vibration into electrical energy, then the charge accumulates on the input capacitor $C_{\mathrm{store}}$ through a full-wave rectifier bridge and the voltage of V_IN_ (voltage on $C_{\mathrm{store}}$) gradually rises. When V_IN_ rises to the high threshold voltage of the undervoltage lockout circuit, the DC-DC voltage regulator turn on and transfer charge from the input capacitor to the output capacitor $C_{\mathrm{out}}$. When the voltage V_IN_ drops to the low threshold voltage of undervoltage, the DC-DC voltage regulator turn off until the voltage of V_IN_ rise up to the high threshold voltage of undervoltage lockout again. This cycle can achieve periodic, intermittent energy storage. The final size of the board is 28.9 mm × 21.0 mm × 1.4 mm.

The experimental results show that the powered management circuit can meet the voltage, current, and power consumption needs of a wireless sensor node. As shown in Figure 7b, when the piezoelectric energy harvester starts working, the storing capacitor is charged, the output voltage rises stepwise, and finally stable at 3.585 V.

## 4. Experimental and Signal Processing

**Figure 8:**
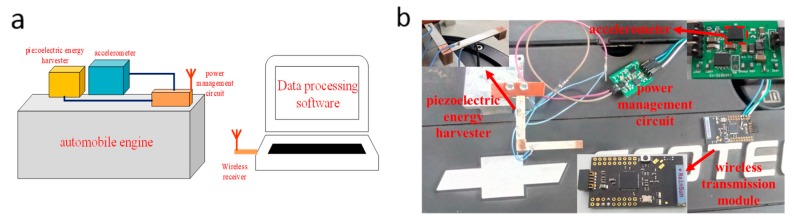
(**a**) Schematic diagram of test platform installation; (**b**) The picture of experimental test system.

The schematic diagram of the test platform construction is shown in Figure 8a, mainly including: (a) an automobile engine; (b) a piezoelectric energy harvester; (c) a power management circuit; (d) an accelerometer; (e) a wireless transmission module. The subject of this experiment is the Chevrolet Epica 2HO automotive engine. The accelerometer selected for this test is a 3-axis, MEMS accelerometer ADXL343 (Analog Devices, Inc., Norwood, MA, USA) with digital output of 13 bits and its measurement range is ±16 g. Figure 8b is the picture of experimental test system.

To process the complex vibration signals, this paper choose MATLAB R2015a (MathWorks, Natick, MA, USA) to analyze and process the data. Since if the speed of the engine is too low, the fuel will not burn enough in the cylinder and intensify engine carbon accumulation. And if the speed is too high, overheating of the temperature will cause a great burden on the engine and bring great wear on it. Therefore, the vibration signal from the *z*-axis at the 2000 r/min, 2500 r/min and 3000 r/min were extracted to process in this paper.

**Figure 9:**
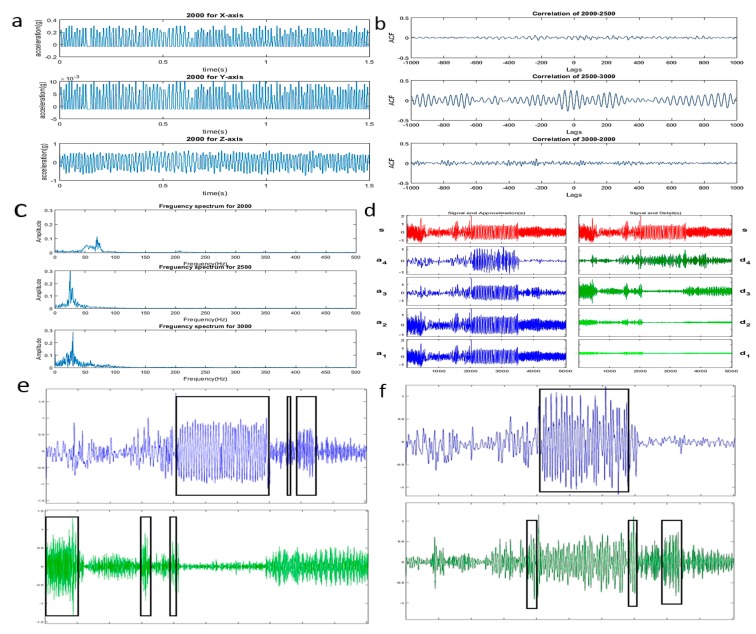
(**a**) The initial vibration signal of 2000 r/min, 2500 r/min, and 3000 r/min; (**b**) cross correlation analysis of the three sets of signals; (**c**) spectrum of the three sets of signals; (**d**) four level wavelet decomposition of the vibration signals; (**e**) the third level wavelet decomposition, upper (0 Hz–62.5 Hz), lower (62.5 Hz–125 Hz); (**f**) the forth level wavelet decomposition, upper (0 Hz–31.25 Hz), lower (31.25 Hz–62.5 Hz).

The time domain diagram of the acquired signal are shown in Figure 9a. It can be seen that the time domain diagram of the signals are smooth. It can initially be concluded that the engine is running in a healthy state.

In order to analyze the similarity between vibration signals of different speeds, cross-correlation analysis was used and the results is shown as Figure 9b. The horizontal axis Lags represents the delay and the vertical axis ACF represents the normalized autocorrelation coefficient. It can be clearly seen that the cross-correlation coefficient between the speed of 2000 r/min with 2500 r/min and 2000 r/min with 3000 r/min basically trends to zero, and there is a relatively weak correlation between the speed of 2500 r/min and 3000 r/min (the correlation coefficient is approximately 0.2). It can be concluded that the three sets of signals did not correlate significantly, which indicates that the signals at different frequencies can be easily distinguished.

Since the signal of each component contained therein can be better observed in the frequency domain, the frequency structure of the signal needs to be further analyzed. The vibration frequency of the vibration signal is closely related to the speed of the engine and many fault signals have a frequency distribution below the rotational frequency or a non-integer multiple of the rotational frequency. Therefore, paying attention to the frequency of these places can help find engine fault information. The spectrum of the vibration signals at speed of 2000 r/min, 2500 r/min, and 3000 r/min are given in Figure 9c. The spectrogram of the vibration signal with a speed of 2500 r/min and 3000 r/min has almost no extra frequency components, but the frequency signal spectrum of the speed 2000 r/min has some other frequency components besides its rotation frequency, that may be caused by insufficient combustion of the fuel in engine of low speed.

In many cases, the engine vibration signal is not a stationary signal. The phase or frequency of these signals changes with time and local analysis of signals is needed. Wavelet analysis was used to analysis the signal and the analysis results are shown in Figure 9d.

Among them, *s* is the original signal, *a* represents the low frequency part of the signal and *d* represents the high frequency part of the signal, and the number of the angle mark represents the number of levels of wavelet decomposition. Since the frequency of the signal is concentrated at 20 Hz–100 Hz, it can be seen in the figure that *d*_1_ and *d*_2_ are substantially near zero, that is, there are almost no frequency components greater than 125 Hz. Further analysis of the third and fourth layers is shown in Figure 9e,f. It can be seen from the figure that the components with the frequency of 0 Hz–62.5 Hz mainly occur in 2.1 s–3.5 s, 3.7 s, and 3.9 s–4.2 s (black frame in the figure); the components with the frequency of 62.5 Hz–125 Hz mainly occur in 0 s–0.5 s, 1.5 s–1.6 s, and 1.9 s–2.1 s and there are more frequency components at low frequencies. It can be further seen from Figure 9f that the frequency components of 0 Hz–31.25 Hz are concentrated in 2.1 s–3.4 s, which show that the speed transition point of the engine is at 2.1 s and 3.4 s and the engine may breakdown at this time period.

## 5. Conclusions

In this paper, a complete battery-free, self-powered engine health monitoring solution is proposed. The experimental results show that the proposed wideband piezoelectric energy harvester designed by this paper can provide enough energy for the system. The analysis of the collected signal also shows that the engine vibration signal can be used as an effective basis for engine health monitoring. The system makes a meaningful exploration for the mature application of self-powered wireless engine health monitoring systems.

## Figures and Tables

**Table 1:** The eigenvalues of vibration signal in Z direction on the cylinder head of the 2HO engine.

Speed (r/min)	Acceleration Amplitude (m/s^2^)	Acceleration Frequency (Hz)
800	−0.49~0.58	51.88
1500	−0.76~0.98	53.41
2000	−1.63~1.76	74.77
2500	−2.30~2.32	94.60
3000	−3.45~4.60	111.39
3500	−5.97~5.95	129.70
4000	−6.67~6.10	132.75

**Table 2:** The dimensions of the micro wideband piezoelectric vibration energy harvester.

Parameter	Beam_1	Parameter	Beam_2
Length of the Beam_1 Lb1 (mm)	28	Length of the Beam_2 Lb2 (mm)	30
Width of the Beam_1 Wb1 (mm)	5.5	Width of the Beam_2 Wb2 (mm)	5
Thickness of the piezoelectric layer tp1 (mm)	0.25	Thickness of the piezoelectric layer tp2 (mm)	0.25
Thickness of the substrate layer tb1 (mm)	0.25	Thickness of the substrate layer tb2 (mm)	0.25
Mass_1 (g)	2.90	Mass_2 (g)	1.37
